# Barriers to the implementation, uptake and scaling up of the healthy plate model among regular street food consumers: a qualitative inquiry in Dar-es-Salaam city, Tanzania

**DOI:** 10.1186/s40795-022-00589-6

**Published:** 2022-10-06

**Authors:** Gibson B. Kagaruki, Michael J. Mahande, Katharina S. Kreppel, Doris Mbata, Andrew M. Kilale, Elizabeth H. Shayo, Sayoki G. Mfinanga, Bassirou Bonfoh

**Affiliations:** 1 Research Programs, National Institute for Medical Research, Tukuyu Medical Research Centre, Tukuyu, Box 538, Mbeya, Tanzania; 2 Department of Epidemiology & Biostatistics, Institute of Public Health, Kilimanjaro Christian Medical University College, Box 2240, Moshi, Tanzania; 3 School of Life Science and Bioengineering, the Nelson Mandela African Institution of Science and Technology, Box 447, Arusha, Tanzania; 4 Department of Public Health, Institute of Tropical Medicine, Antwerp Belgium, Antwerp, Belgium; 5 Research Programs, National Institute for Medical Research, Headquarters, Box 9653, Dar es Salaam, Tanzania; 6 Research Programs, National Institute for Medical Research, Muhimbili Centre, Box 3436, Dar es Salaam, Tanzania; 7 Department of Epidemiology & Biostatistics, Public Health Muhimbili University of Health and Allied Sciences, Box 65001, Dar es Salaam, Tanzania; 8 Departiment of Research and Development, Centre Suisse de Recherches Scientifiques en Côte d’Ivoir, Abidjan, Côte d’Ivoire

**Keywords:** Street food, Street food vendors, Street food consumers, Healthy plate model, NCDs/diabetes

## Abstract

**Introduction:**

The healthy plate model is considered one of the practical approaches to reduce the average portion of staple food in main meals, consequently reducing the risks associated with diabetes and other Non-communicable Diseases (NCDs). Despite its potential benefits, studies on the implementation of the healthy plate model are limited in Africa. An inquiry explored barriers to implementation, uptake, and scaling up of the healthy plate model among street food vendors and consumers in three districts of Dar-es-Salaam city in Tanzania.

**Methods:**

A qualitative research design was adopted. Qualitative data collection techniques were employed including; Key Informant Interviews (KIIs) with purposefully selected food and nutrition stakeholders at the National, Regional, District and Ward levels. Focus Group Discussions (FGDs) were conducted with purposefully selected street food consumers and vendors. A total of (13) KIIs were conducted as well as (6) FGDs with street food vendors (2 FGDs) and consumers (4 FGDs). Interview data was managed using Nvivo 12 Software and analyzed thematically.

**Results:**

Three key themes emerged from participants’ accounts: (i) strategic policy barriers, (ii) food production and preparation environment barriers (producers and vendors), and (iii) individual barriers (consumers and vendors). The strategic policy barriers included absence of guidelines and regulations that focus on NCDs linked to nutrition and lack of education guidance for vendors and consumers. The food production and preparation environment barriers included safety and risks concerns regarding the quality of water used for irrigation and washing fruits and vegetables and the areas where vegetables and fruits are grown and prepared. Individual barriers included low consumer income, knowledge on nutrition, unhealthy eating practices and; low vendors’ knowledge as well as low investment capital.

**Conclusion:**

Implementation, uptake and scaling up of the healthy plate model for street food consumers in Dar es Salaam City continues to be constrained by barriers in policy, food production and preparation environment, and individual obstacles. Strengthening of food and nutrition policies, ensuring safety of the food production and preparation environment and, consumer and vendor awareness creation and income generation efforts, provide useful entry points for the successful scaling up of a healthy plate model. This could consequently contribute towards prevention of diet related NCDs, including diabetes.

**Supplementary Information:**

The online version contains supplementary material available at 10.1186/s40795-022-00589-6.

## Introduction

Non-communicable diseases (NCDs), including diabetes, are a growing public health problem globally [1–3]. In 2016 alone, NCDs were responsible for an estimated 40·5 million (71%) of the 56·9 million deaths recorded worldwide, with most of these deaths (42%) occurring among people aged below 70 years [4, 5]. The risk of dying from NCDs is high in low and middle-income countries, especially in sub-Saharan Africa and Asia [4, 5]. Diabetes is among the most prevalent NCDs that continue to contribute to this excess morbidity and mortality. The prevalence of diabetes at a global level was 9.3% in 2019, slightly higher in urban contexts (10.8%) than rural settings (7.2%) [6]. Estimates indicate that the number of people with diabetes will increase from 578 million in 2019 to 700 million in 2045, if adequate mitigation strategies are not established or strengthened and implemented [5].

Various efforts to address the burden of NCDs, including diabetes, are in place at global, regional and national levels. These efforts include the Global Action Plan for the Prevention and Control of NCDs and the Sustainable Development Goals (SDGs) [5]. The SDGs goal number 3.4 seeks to reduce pre-mature mortality from NCDs by one third by 2030 through prevention and treatment, and to promote mental health and wellbeing [4–7]. Consequently and practically, the WHO recommends the consumption of 400gm of fruits and vegetables daily, an amount considered adequate to protect an individual from increased risks of NCDs, including Type 2 Diabetes [8]. Tanzania developed a strategic plan for NCDs for 2016–2020 [5, 8] and, institutions dealing with food quality control and safety, including the Tanzania Bureau of Standards (TBS), Tanzania Food and Nutrition Center (TFNC) and Tanzania Medicines & Medical Devices Authority (TMDA), were established [9–11]. These efforts are geared towards reducing NCDs/Diabetes risk factors including physical inactivity, unhealthy diet, harmful alcohol consumption, and smoking tobacco products among others [5, 12–14].

Despite these strategic efforts, the burden of NCD risks factors, particularly those related to food consumption among urban dwellers, especially street food consumers, remain high. A recent baseline assessment conducted in Dar-es-Salaam among regular street food consumers as part of this study revealed a high magnitude of Diabetes risks including overweight/obesity (63.9%), raised blood pressure (42.5%), raised triglycerides (13.5%) and raised blood glucose (6.6%) [15]. The findings appear to mirror what has been previously documented among urban populations in Tanzania [13, 16, 17].

The street food informal business provides employment to a significant number of people and represents an essential food source for billions of people in many countries. Reports indicate that over 2.5 billion of the global population and about 50% of people in sub-Saharan Africa consume food prepared and sold in streets, including the majority of low and middle-income inhabitants of Dar-es-Salaam City [17, 18]. The previously documented increasing burden of NCD risk factors and Diabetes rates among street food Consumers in Dar-es-Salaam City in different contexts, has informed a range of pilot interventions proven to reduce risks related to unhealthy eating habits [19–21], such as *the healthy plate model*. The healthy plate model is a conceptual and visual aid that guides consumers towards healthy eating habits by showing the recommended portions and types of food groups or drink that make up a healthy and balanced diet [20]. This plate was designed and validated by nutrition experts from the Harvard School of Public Health [20–22]. Half of each plate is made up of vegetables and fruits, ¼ of whole grains as source of energy and ¼ of protein. The dish is prepared with healthy plant oils and accompanied by drinking water or milk. The model is considered one of the practical approaches to reduce the average portion of staple food in main meals, consequently maximising the intake of vegetables and protein rich foods [23]. If implemented successfully, the model has the potential to promote healthy eating by reducing dietary calorie intake and redistributing macronutrients, consequently reducing the risks associated with Diabetes and other NCDs [22–25]. Despite its potential benefits, studies on the implementation of the healthy plate as an entry point to the prevention of Diabetes and other NCDs are limited in Africa. To address this knowledge gap, this study explored the barriers that could impede the implementation, uptake and scaling up of the healthy plate model in Dar es-Salaam City, Tanzania. The study sought to generate an understanding of challenges faced by street food vendors, customers and the food and nutrition system, which may prevent the scalability of the healthy plate model in the city. The findings are expected to provide useful practical and policy insights for successful intervention implementation targeting vendors and consumers of street food as a strategic approach to reducing Diabetes risks in the country.

## Methods

### Study design

The inquiry employed a qualitative research design that facilitated tapping into perceptions and experiences of street food vendors and consumers as well as food and nutrition administrators. A qualitative research design [26] was used to answer a broad question of *what* the barriers for implementation, uptake and scalability of a healthy plate model are. This approach sought to develop an understanding and describe phenomena (e.g., barriers for implementation, uptake and scalability of a healthy plate model) without necessarily testing an existing theory [26]. This approach offers an effective way of gathering a deeper and richer contextualised understanding of research participants’ perceptions and perspectives [26–29]. It takes into consideration the experiences of a healthy plate model from the point of view of regular street food vendors, consumers and food nutrition administrators. In our study context, these experiences may differ from providers in other contexts, with regard to their expectations, resources and policy and regulatory environments.

### Study setting

The study setting has been previously described in detail in our baseline survey [30]. Briefly, the study was conducted as part of a larger nutritional intervention cluster randomized field trial aiming to assess effectiveness of interventions to reduce cardio-metabolic risk factors among regular street food consumers (RSFCs) in Dar es Salaam, Tanzania. Dar es Salaam, as one of the fastest growing urban centres in Africa [31, 32] and with a large population dependent on street food [16–18], the city presented an ideal study site. High numbers of street food vendors and a large consumer population formed an important selection criterion of this city, as was the presence of the headquarters of all the country’s food regulatory authorities. This included the Tanzania Food and Nutrition Centres (TFNC), Tanzania Bureau Standards (TBS) and Tanzania Medicines & Medical Devices Authority (TMDA) during the time of the study and facilitated access to administrators in these offices.Within Dar es Salaam City, the study was conducted in three selected districts that were part of the baseline study preceding this inquiry [30]. In each district, two street market sites were chosen for the study. These included Kawe and Tandale in the Kinondoni district, Mbezi and Manzese in the Ubungo district and Ilala Boma and Kisutu in the Ilala district.

### Study population

The study participants included purposefully selected street food vendors, consumers, key food nutrition administrators, including ward and district health environmental officers, and district TDMA coordinators, as well as focal persons from TFNC, TBS and TMDA (national level).

### Participants’ enrollment and sampling

To explore the barriers to implementation, uptake and scaling up the healthy plate model, vendors and consumers in street food markets were purposely selected for FGDs. Food vendors were recruited through their market managers and leaders. The selection criteria included being aged 18 or above; selling food at the current site for at least 12 months; having at least seven customers who have been consuming at least three lunch meals per week for at least 3 months. Being ready to implement the components required for an intervention and being ready to continue vending food at the same site for at least another 12 months were other eligibility criteria. Regular street food consumers were eligible for the study if they met the following criteria: being aged 25–64 years; consuming at least three lunches per week at the same street food vendor; having no plans to move out of the study area in the next 12 months and have been consuming street food for not less than a year. More details on the selection of participants can be found elsewhere [30]. Based on expert opinion, the authors then purposefully selected 13 food and nutrition stakeholders for KIIs. Written invites were sent to the health environmental officers at ward and district levels, to one regional nutritional officer, TMDA staff at district and national levels and to TBS and TFNC staff at national level.

### Data collection tools

A consultative process involving experts at the National Institute for Medical Research, the Kilimanjaro Christian Medical University College, and the Nelson Mandela African Institution of Science and Technology, as well as the Sokoine University of Agriculture and the Muhimbili University of Health and Allied Sciences was employed to generate and translate KIIs and FGD guides into Swahili. These guides were then translated back to English and checked for conceptual equivalence. Questions in the KII guide ranged from perceived magnitude and trend of NCDs/Diabetes and their risk factors, challenges that impede supply and demand of a balanced diet in the street food business, to issues hindering the enforcement of existing policies and regulations. Likewise, questions in the FGD guide ranged from awareness of Diabetes risk factors to issues hindering or promoting consumption of healthy foods/a balanced diet among street food consumers. Three research assistants fluent in Swahili language were trained on the use of data collection tools and techniques for this study. Close and supportive supervision was done throughout data collection and at the analysis stage to ensure data quality. The guides (see Appendix A) were tested in purposefully selected settings and refined accordingly.

### Duration of the study

This qualitative inquiry study was carried out between July and September 2018 in Tanzania.

### Data collection process

Both KIIs and FGDs were conducted in a quiet and isolated room entirely disconnected from regular activities. Upon arrival, research assistants obtained informed consent and engaged respondents for approximately 60 min in a semi-structured dialogue on the study content (data collection tool). Each KII was conducted by one research assistant and two research assistants held FGDs face-to-face. All interview sessions were audio recorded. In addition, field notes were taken to ensure all key findings and fieldwork descriptions were captured. Thirteen (13) KIIs and six (6) FGDs were conducted (2FGDs for vendors and 4FGDs for consumers), provided enough information to achieve data saturation [33, 34]. The data was collected following all the research ethics, which provides a window for replication and validation, by other researchers.

### Data management and analysis

Audio recordings were transcribed verbatim and translated by research assistants. The transcripts were then verified by a social scientist who was part of the research team by listening to the audios to confirm the correctness of the transcriptions. After transcription and translation, interview transcripts were de-identified, pseudonyms generated for each participant and the data uploaded into NVivo 12 software (QSR International, Australia) for deductive and semi-inductive thematic coding.

Thematic analysis embraced the Braun and Clarke [28] approach. More specifically, two social scientists examined the research questions and interview guides and generated a list of initial themes and subthemes. The research team reviewed these themes and subthemes that formed the coding framework before including them in NVivo 12 software (QSR International, Australia) nodes and sub nodes. In the reviewing process, the research team went through few transcripts to confirm the correctness of the themes and sub themes and refining was done as necessary and until a consensus was reached within the team [35]. However, open coding was allowed for new information that emerged as the coding process progressed. The emerging codes were sorted into existing subthemes and themes, followed by a collation of all relevant coded data extracts within identified themes [36]. Then, the coded contents were exported from NVivo 12 software (QSR International, Australia) to MS excel sheet matrix document for analysis and interpretation. Analysis focused on drivers for the uptake and scaling up of the healthy plate model as they manifested in a specific context (Dar es Salaam city of Tanzania) but it was preceded by documenting the perceived NCDs trend in their residences, awareness and perception of study participants on the risks factors for diabetes. To ensure Trustworthiness and reflexibility, two components include validity and reliability were considered during data collection and analysis. Validity of the results was achieved through day-to-day members checking through reporting and meetings, it was mainly done during data collection and later during data analysis [36]. Validity was also achieved through triangulation of results collected using key informant interviews involving informants who were purposively selected from different food regulatory bodies (TBS, TFNC and TMDA) and administrative levels (ward, district, regional and national level) as well as focus group discussion involving street food consumers and vendors. Furthermore, both reliability and validity were achieved through use of coding system. The codes were developed in relation to the basic themes, organizing themes and ultimately the general theme [37], see Table 1.

**Table 1:** Thematic analysis of data

Codes	Basic themes	Organizing themes	General theme
… When you put vegetables, they do not eat	Low consumption of fruits and vegetables	Individual barriers	Barriers to the implementation, uptake and scaling up of the healthy plate model
…Sometime the issue is not vendors’ capital, it all depends on consumer’s food preference			
…. The interest of food vendors is to maximize profit rather than safety and quality			
….. Low capital lead food vendors to buy cheap foods and unsafe cooking oil			
……..Fruits are expensive and normally are sold separately, thus those with high income can afford buying them			
……Due to food preference, some consumers say the food is meat not vegetables			
…..Vegetables are eaten by poor people			
…Due to low awareness of balanced diet, the desire is stomach get full			
….I am from Changha tribe, I have never seen my grandfather eating vegetables			
….Due to low capital the vendors get cooking materials on credits and pay the shops owners after selling			
….We are ready to offer the required food groups provided they are needed by customers	Food vendor’s readiness to provide health plate		
…Demand or preference always shape the supply			
……With your small amount of money, you are given the food according to your money with less vegetable or not at all			
…The capital of vendors is low, the government should provide loans specific for vendors			
…..…Vendors tend to grade their customers according to their income i.e. those with low, middle and high income then they prepare the food based on the level of consumers’ income			
….Some vegetables are irrigated with water from sewage systems and factories, thus people who are aware of production environment are discouraged to consume vegetables	Unsafe Environment for production of Vegetables	Food production and preparation environment barriers	
…..The vegetables that are being grown, along the main road are polluted with vehicle smoke and dust			
…..Some of the vegetables and fruits growers spray insecticides and harvest within a short time and take them to the markets			
….Price of fruits will depend on seasons and distance from the sources	Vegetables price variation		
…..Vegetables irrigated with water from valleys rivers are cheap while those irrigated with tape water are expensive			
…..You can’t compare the prices of healthy and normal plate, obvious, the former is expensive			
…. Most of the time they are oily food and poor-quality oil	Poor quality of preparation of fruits and vegetables		
….….Consumers may fail to consume vegetables because they do not trust the production and preparation environment			
…. Food quality starts from production, and transportation to processing			
….Sometimes water used to wash vegetables/fruits before cooking is unsafe			
….You find some cooked vegetables have sands not fit for eating			
…..The regulations should start with food vendors that the food provided should be balanced diet	Absence of guidelines and regulations on the amount and types of food groups to be served/meal	Strategic policy barriers	
…..Food vendors are not registered and are emerging everyday thus it is hard to regulate them and organizing them for training			
…. the focus of regulatory bodies is on food safety and not on the amount of food groups served and consumed by customers			
… In absence of guidelines it is difficult to implement food quality measures			
……The government should prevent people from cultivating vegetables in contaminated areas			
….Instituting an healthy plate is policy issue			

## Results

### Participants’ demographic characteristics

Six FGDs were conducted of which two were with food vendors and four with regular street food consumers. Participants were recruited from different sites in Kinondoni, Ilala and Ubungo Municipalities. Each FGD had 8–12 participants including males and females aged between 25 and 64 years. In total 61 participants took part in the six FGDs of which the majority had primary and secondary school education. A total of 13 male and female key informants were recruited for interviews from ward, municipal, regional and national levels. These included environmental officers and food and drug coordinators at ward and municipal levels, nutritionists at regional level, TMDA representatives at municipal levels, and representatives from TBS, TFNC and TMDA at the national level.

#### Awareness and perceived magnitude of NCD risks in the community

Awareness and perception of the food administrators, consumers and food vendors regarding the magnitude and trend of NCDs/Diabetes risks were explored during this study. The aim was to understand their awareness and perception on the matters. This included their views on NCDs risk behaviours related to type 2 diabetes such as unhealthy eating habit, harmful alcohol consumption, smoking, physical inactivity etc. as well as NCDs/Diabetes risks related to physical measurements such as raised blood pressure, overweight/obese etc. in their areas of administration or residence. The study wanted to understand people’s perceived behaviour and physical measurements risks associated with type2 Diabetes. It was affirmed by participants that the risk trend is increasing in urban environment and that there are many lifestyle risks associated with NCDs including type2 Diabetes. They mentioned unhealthy eating habits, alcohol consumption and physical inactivity as common NCDs/type2 Diabetes risks factors in their communities. Other mentioned overweight/obesity as the prevailing NCDs/Diabetes risk factor in their community. Some food and nutrition administrators indicated that urban dwellers have been consuming less natural food, because of the cultural embedded preference of processed foods, which are rich in fat and starch, and contain only little amount of vegetables. Increased personal preference to unhealthy processed food, alcohol consumption and physical inactivity some key informants commented:

*“NCDs/type2 Diabetes risk behaviour is something that is gaining momentum due to the lifestyle that people live here in the city. If you look at the people in the city, most of them are from areas outside the city. They come to the city to do various activities to earn a living in various ways, it is their lifestyle that forces them into such a dangerous situation….surprisingly in our society, such foods from the groups of vegetables and fruits we see as complementary foods, i.e., a person may decide to eat them or not” (KII/Ilala Municipal)*

They went further arguing that people who are working in the office because of the life style they are living, even when you look by eye, it is very obvious that most of them are obese. This is one of the risk factor for type 2.

*“If we do a simple assessment here in the office for the staff in this building, and just measure the height and weight to get a person's BMI, this one here will fall into the overweight category and this one is already obese” (KII/Ilala Municipal).*

Unhealthy eating is increasingly referred as the key risk factor for NCDs and other related conditions, because of the nature of food that are consumed. It is very unfortunate that people are rarely aware that, the food rich in fat and carbohydrate contributes to the most of NCDs, it is even worse that they rarely do exercise as it was expressed by this KI.

*“Risks that can lead to the onset of Diabetes are increasing in urban areas. Unhealthy eating and alcohol drinking habits are the common NCDs risks. For example, most people prefer foods that are rich in fat and carbohydrate and at the same time, alcohol is sold almost in every shop and bar close by. Unfortunately, people do not realize that these kinds of foods cause many non-communicable diseases such as Diabetes. Then the people find that they have gained a lot of weight after eating that unhealthy food and there is no habit of exercise” (KII/ Ward Level Ubungo Municipal).*

#### Definition of healthy plate model according to respondents

Different opinions emerged on what a health plate is and should contain to prevent NCDs including Diabetes. Most of the food vendors were of the opinion that a healthy plate should have less starch, more fruits and vegetables. Some suggested that in their cooking, they should reduce the amount of oil and salt when they cook foods. However, they were not sure what exactly a portion of starch, vegetables and fruits served per person per meal/plate should be. Some of the food vendors had idea on what to eat for a healthy person and what for a Diabetes patient and they commented:

*“To accompany “ugali” (*a traditional staple made from corn flour*), a quarter of the plate should be a piece of fish and the rest a mix of fruits such as mango, watermelon and banana” (FGD/ Food Vendors/Kisutu).*

##### Others reached a consensus that;

*“Consumers should eat a portion that is measured, on average, but every meal that a diabetic person eats should have lots of vegetables, boiled without oil” (FGD/Food Vendors/Mbezi)*

Similar to food vendors, consumers also appeared to be aware of what a healthy plate should look like, however, they as well expressed uncertainties of the exact amount of starch, fruits, protein and vegetables should a healthy plate contains. As it is substantiated with the following quotes:

*“In a meal of rice or ugali, three quarters of the plate should be mixed vegetables, and fruits such as a piece of mango, a piece of pawpaw, and a piece of pineapple”(FGD/Food Consumers/Kisutu)*

##### For others it was explained;

*“A meal should have a small amount of starch, a large amount of fruits, and a large amount of vegetables, say, half- half” (FGD/Food Consumers/Tandale)*

Likewise, the opinions of food and nutrition administrators did not differ strongly from those of food vendors and Consumers. However, they went further to discuss the portion provided by food vendors to their customers. They were of the view that the amount of starch on a plate is higher than the amount of protein, fruits and vegetables and that, sometimes vegetables and fruits are not served at all. Some administrators commented:)

*“The reality is, sometimes when we are at work, we also eat those foods, you find that carbohydrates are served in large portions while protein is served in a small portion the food group that is served in large quantities/amounts is starch and very often a portion of rice or ugali or banana is served with beans or meat. Food vendors do not serve portions of fruit; it does not exist at all. Vegetables can be present in small quantities” (KII/National Level)*

##### Another opinion was;

*“Most of the time vegetables are served in a very small amount, but a person will get sauce, a good amount of carbohydrates, a little protein like meat, but no fruit at all (KII/Health Officer/Kawe)*

### Barriers for uptake and scaling up the healthy plate model

Forming the primary focus of this inquiry, the authors then went ahead to specifically examine the barriers to the implementation, uptake, and scaling up of the healthy plate model. Three key themes emerged from participants’ accounts: (i) individual barriers, (ii) food production and preparation environment barriers and (iii) strategic policy barriers. Table 1 shows a list of codes, basic themes, organizing themes and general theme generated after conducting thematic analysis.

## Individual barriers for implementation, uptake and scale up of a healthy plate model

### Low consumption of fruits and vegetables

Participants suggested that people are not consuming the recommended amount of fruits and vegetables because they are culturally considered as optional and not part of a meal and that people only consume them whenever they feel like doing it. The findings show that it is not a custom whatsoever for the meal of some people in our society to contain fruits and vegetables. They are either considered as luxury or rather between meals or snacks. This suggest that community members should be educated on the importance of including fruits and vegetable in their meals. One food and nutrition administrator commented:

*“Something worse for our society; such foods as vegetables and fruits we consider to be complementary foods, you just eat them when you feel like it. One finds it not very necessary so most of us do not eat them for the sake of improving our health, most of us eat them for the sake of self-satisfaction i.e. treated as snacks” (KII/Kinondoni Municipal).*

#### Similarly, it was explained that;

*“When we go for field supervision of the street food vendors with our ward environmental officers, we normally see the types of food they prepare, they prepare according to their amount of capital and the demand of their customers. Therefore, if a vendor will not prepare according to the preference and purchasing power of their clients then the clients will shift to colleague vendors, therefore, they are forced to comply with that situation to avoid losing clients and income…..” (*KII/, Ilala municipal level)

### Food vendor’s readiness to provide health plate

Furthermore, food vendors reported readiness to provide a healthy plate. However, they were concerned that due to food eating preference some of their customers would not agree with the amounts of each food type provided in each plate unless they have been made aware. Similarly, food vendors noted that some customers do not generally like vegetables. It was cited common for example to find a customer has eaten all the food (protein and carbohydrate) but left behind the vegetables. However, the findings show that despite other challenges faced by the food vendors, they are ready to serve small portions of fruits and vegetables. It was noted that;

*“Carbohydrates are served in abundance compared to vegetables; you may find that the amount of vegetables served is too small and the amount of meat may be more than the vegetables because it attracts consumers. Most people like to eat to fill their stomach and due to low income, the majority ends up eating an unbalanced diet with large amounts of carbohydrates. Frankly speaking, vegetables and fruits are rarely served, for example when you go to “Mama Lishe”, you will find the pots used to cook ugali and rice are bigger than those used to cook vegetables are. In most case, no fruits are served, unless there is another vendor who sells them separately and not as a portion of an ideal food plate” (KII/National Level). *Interestingly, in one of the FGDs it was explained that;

*“Based on my experience, our clients will opt for the plate with no vegetables and fruits since it is cheaper. For the healthy plate, only one out of ten people will opt for it, those who know the importance of balanced diet”* (FGD/Food Vendors/Tandale).

### Financial constraints

In addition, financial concerns emerged because the amount of food type in each plate will consequently rise the price to cover the expense of fruits and vegetables. Financial issue was also linked to the capital of food vendors. Vendors commented that their capital is meagre, therefore, cannot always afford the price of fruits. Some, however, suggested that it is possible to include vegetables and fruits in some seasons when the price of these products is low at the markets. Furthermore, the meagre capital of street food vendors force them also to use unhealthy cooking oil which are sold at a cheap price, mainly animal oil. For example, it was explained that;

*“What I can guess is that vendors look at the income of their customers; there are low-income customers, middle-class customers and those who are well off, but since the majority who consume their food have low income, they adjust the quality, value and price of the food they sell to that majority. ….in fact, food vendors’ capital is small, you find many of them going to the store to buy rice on credit to cook and sell; most of them they do not even have the capital to buy and store rice for a week. Instead, they go to the store and buy two kilos of meat and pay for it once they have sold it in the evening;” (KII/Ward Level/ Kinondoni Municipal).*

#### Similarly, it was explained that;

*“Financial problems can affect both customers and food vendors. If the capital is too small it means the vendor is trying to look for cheaper things to serve, so that the customer can get food but the quality is then compromised” (KII/ National Level)*

#### In addition it was explained that;

*“In reality, the capital of the vendors is low; if it is possible I advise that the government should consider providing loans to these street food vendors, I mean the loans that specifically target the street food vendors not the general population to ensure wide access of the loans to food vendors (KII/Ward Level/Kawe).*

#### An extract from the FGD suggests that;

*To my views I can say to get healthy plate depends on the environment where food business is conducted, you can find that in some places a plate sold at Tshs 2500/ (*> *1USD) containing all required food groups. Thus, our food vendors have studied our environment and found that if they prepare and sell a plate at Tshs 2500/, who will buy it?* (FGD/food consumers/Tandale).

In association with the financial constraints, the consumers further argued that it would be difficult for food vendors to provide them with a healthy plate because their capital is too small to afford the price of vegetable and fruits. However, it was noted that, there are other places that sell food and provide vegetables and fruits but the price is too high for them to afford, therefore, they choose to go to vendors offering affordable meal. Food and nutrition administrators’ descriptions highly reflected vendors and consumers’ concerns.

#### For example in one of the FGDs with consumers, it was argued that:

*.....The street food vendors prepare the food based on their capital and have to minimize the running costs to make profit, therefore, it is difficult to get a balanced diet from street food vendor due such circumstance*” (FGD/Mbezi/consumers)

*“*Based* on my experience, our clients will opt for the plate with no vegetables and fruits since it is cheap. For the healthy plate, only one out of ten people will opt for it, those who know the importance of balanced diet” (FGD/Food vendors/Tandale).*

### Consumers’ consumption cultural habits

The other challenge cited is the cultural habit of non-preference of vegetables. It appears that sometimes consumers are given plates including vegetables, even if in small amounts, but they often leave out the vegetables. At the heart of low consumption of vegetables was the concern of conscientiousness of both food vendor and consumers on the importance of a balanced diet in their meals. Diet education for consumers and food vendors was suggested as a strategy to address this. One food and nutrition administrator commented:

*…Cultural and food preference habits are among the challenges. You find someone has received a plate with ugali, vegetables and meat, that person may eat all ugali and meat and leave aside the vegetables because he is not used to consume vegetables from childhood…” (KII/National Level)*

In line with the quote above, it was also explained that;

*“I see the biggest thing that could be affecting is the lack of dietary education, understanding that eating this will help me in that way. To address this, we think educating Consumers and food vendors is of paramount importance” (KII/Ilala Municipal).*

## Food production and preparation environment barriers for healthy plate model adoption

Another challenge related to uptake and scaling up of the healthy plate model emerging from participants was about where food is produced and prepared. This is because provision of quality food depends on the source and/or environment where food is grown and how it was prepared.

### Production of vegetables and the environment

The issue of poor food production and preparation environment emerged during discussions with key informants. Most participants suggested that food quality is highly dependent on the quality and safety of environment where it is grown. Fears and concerns of contaminated environment emerges as among the possible reasons for people not consuming vegetables in Dar es Salaam city. This is much related to what people see or the information people obtain from friends, relatives and the streets about the source of where the foods are grown. Some food and nutrition administrators commented:

*“The environment where crops are grown is very important because the quality of the food starts there; it goes from cultivating, producing, and processing and finally reaches the Consumer. Now if the environment is not good the effects are huge to the producer and the consumer” (KII/Kinondoni Municipal)*

Places where this vegetable are grown determine the safety, there are places in Dar es Salaam that are known for growing vegetables, however, the water that is used for watering are said to be contaminated. This allegation influences the use of vegetables that comes from those areas. Some food and nutrition administrators commented:

*“People talk about, for example, the amaranth ‘Mchicha’ grown in Magomeni, Kigogo, Mismbazi etc. They say that it is contaminated with dirty water from the canals which is used for irrigation there, so we can't eat vegetables from Manzese, Magomeni, Msimbazi; we eat amaranth ‘Mchicha’ from Mbezi*” (*KII/ Ilala Municipal*).

In addition to the above explanations, it was also noted from the health officer that;

*“That is now a very big challenge. A large percentage of the people here in the city are scared; if you talk to people, they tell you it is dangerous to eat vegetables because they are grown in dangerous environments. So, one thinks it is better to skip vegetables, because they are not safe because they are irrigated with dirty or polluted water”* (KII/Health Officer/Ilala Municipal).

### Production and price of the vegetables

The environment that vegetables are grown or prepared were also said to affect price as well. It is common to find bunches of Amaranth ‘Mchicha’ of the same size sold at different prices because they were watered with either safe (tap water) or unsafe (valley, where waste is discharged) water. One food and nutrition administrator commented: *“Right now, for a bunch of amaranths you may find the seller is telling you this is two hundred and this is six hundred Tshs, for the same size. If you ask him/her “why you are selling this for six hundred Tshs?”, he will tell you I use tap water to water it, while the bunch costing two hundred Tshs is grown and watered with dirty water from the valleys where waste is discharged”* (KII/ Ilala Municipal)

### Quality of preparation of fruits and vegetables

In addition, the quality of water used to wash the fruits before being served emerged as the concern because the water was considered to be unsafe (contaminated or not boiled) thus exposing consumers into fecal–oral route diseases such as cholera, hepatitis A, hepatitis E, cholera, adenovirus, and E. coli. For example, one of the food nutrition administrator commended:

*“When at home, you can wash your vegetables and fruits with clean and safe water. However, when you come here at the food vendor’s place you may find sand within the fruits and vegetables. What they do is just cooking (FGD/Consumers/Mbezi)*

*… You find someone has received a plate with ugali, vegetables, some fruits and meat, that person may eat all ugali and meat and leave aside the vegetables and fruits because he/she not trusting the environment where vegetables are grown or quality of water used to wash fruits” (KII/National Level).*

## Strategic policy barriers to a healthy plate model adoption

A recurrent barrier facing food quality control institutions emerging in participants’ descriptions are the concerns of the absence of guidelines and regulations on the amount and types of food groups to be served in one meal. Participants were of the opinion that a priority is often given to whether the food is safe for the consumers. Some participants, for instance health officers, were asked to focus on food hygiene, safety and quality in terms of preparation and servings to customers. Some institutions, for instance TMDA, were advised to focus on food safety and quality but with little focus on nutritional values of the food itself.

### Guidelines and regulations on the amount and types of food groups to be served/meal

A guideline or policy is a plan or explanation to guide one in setting standards or determining a course of action. From the views of the food nutrition administrators it was found that, the concentration of the current guidelines and regulations have been on the harmfulness of the food groups. It was also noted that absence of guideline and regulations that focuses on food nutrition is an obstacle to the uptake and adoption of ideal and health plate. Absence of guidelines and regulations that focuses on nutrition value of food the situation that hinders monitoring of nutrition illness related intervention including uptake and scaling up the health plate model. The focus of the guidelines and regulations is rather on the safety of the food, and not necessarily the quality or quantity.

#### One of the key informants explained that;

*“We have no guidelines requiring us to monitor risks for nutritional illness; our focus is mainly on risks for infectious diseases. Even my immediate supervisor requests from me to monitor and report in that area only. I am also sure that most people are not very aware; hence, with guidelines in place we will be able to inform them that they should eat food in moderation i.e. less carbohydrates, more vegetables, and more fruits. I think the response will then be greater. Then they will be able to tell vendors to serve less carbohydrates and more fruits and vegetables” (KII/Ward Level Kinondoni Municipal).*

*“At this institution [Name hidden] we look at food quality in the sense that the food is not harmful to the consumer, but a balanced diet is not mentioned in the authority guidelines…” (KII/National Level).*

#### Extract from Ilala is;

*It is a big challenge that needs to be addressed because if the guideline containing information on nutrition values of foods does not exist even the implementation will be difficult” (KII/ Health officer Ilala Municipal)*

#### Another food and nutrition administrator commended:

*Our priority is to make sure that hygiene and safety standards are adhered to. For example, if a person is a food vendor, the food is supposed to be clean; the vendor wears an apron and working and selling environments are clean. We also do health routine check-ups and screening of vendors and give them investigation results and a certificate every six months, these are the priorities for our department ……… “We see problems related to food nutritional value, however, our main focus is on food safety and quality, because we are scared of disease outbreaks and infectious diseases such as cholera, for example, hence, our main focus is on the food safety, leaving aside issues of a balanced diet or nutritional values” (KII/Ilala Municipal).*

## Strategic measures to overcome barriers for uptake and scaling up the health plate model

### Education to vendors and consumers

Education is key to any project success. The uptake and scaling up health plate model is not isolated from this. In relation to this study, both customers and food vendors have to be educated on the importance of eating healthy diet. As it stands, during this study it was explicitly explained the need for education;

*Vendors should be educated on how to prepare a balanced diet first; but also, consumers should be educated on the importance of eating a balanced diet, and change their eating habits to avoid the risk of getting Diabetes (KII/Health officer/Ward Level)*

It was also noted that;

*“Most people are not that educated they are very normal people. Thus, we should sensitize them that people should eat food in moderation i.e., less carbohydrates, more vegetables, more fruits I think the response will be greater. Then they will be able to tell vendors maybe put a little ugali and give me fruits and vegetables” (KII/Health officer ward level, Kinondoni Municipal).*

## Discussion

This study was conducted to explore the barriers to the healthy plate model implementation, uptake and scaling up among street food Consumers in Dar-es-Salaam city of Tanzania. The study was designed with an understanding that the majority of people in urban areas of low and middle-income countries, including Dar es Salaam city, continue to rely on street food vendors for their daily meals [18, 38]. This is in the context of increased evidence of heightened risks of NCDs and Diabetes among frequent street food Consumers in different areas. Such heightened risks have formed the basis for an emphasis on the *the healthy plate model* as a practical approach to promote healthy eating by reducing dietary calorie intake and redistribution of macronutrients, consequently reducing the risks associated with Diabetes and other NCDs [22–25]. Our findings bridge the evidence gap in Tanzania by identifying the issues that may be affecting the implementation of the healthy plate as an entry point for the prevention of Diabetes and other NCDs. These findings may be useful to food and nutritional administrators and regulators in creating a more proactive policy framework for successful intervention implementation targeting street food vendors and Consumers aiming at reducing Diabetes risks in the country.

Our findings indicated that the implementation, uptake and scaling up of a healthy plate model is constrained by three complex and multilayers barriers. These include individual barriers, food production and preparation environment barriers and strategic policy barriers. At strategic policy level, the findings indicate the absence of clear guidelines and regulations that focus on nutritional illness risks; the study only found that currently available reference and monitoring practices prioritize the food safety. Likewise, the absence of healthy eating of diet education guideline on healthy food emerged as a common challenge. A guideline or policy is a plan or explanation to guide one in setting standards or determining a course of action [39]. Lack of guidelines or policy in a certain component of health management has potential implications on quality of services and management outcomes [40]. Having no guidelines in place for monitoring illnesses resulting from nutrition prevents the observation of any positive effects after implementing a healthy plate model [41]. The impact of this lack of guidelines prevent health systems in responding to NCDs, including Diabetes, through primary prevention, since people will continue consuming unhealthy because they are not well informed [42, 43]. This will continue if the consumers awareness is not raised, to increase the magnitude of cardio-metabolic risk factors including overweight and obesity, raised blood pressure, raised triglycerides and raised blood glucose which are already high among street food consumers in Dar-es-Salaam city [30].

Previous studies have shown an association between street food consumers eating within or above recommended amount of serving of fruits and vegetables per week and, overweight/obese [30]. Eating less fruits and vegetables is often associated with the interaction between income, practices and knowledge of street food consumers and vendors, which is partly linked to the absence of policy and guidelines to regulate the street food business and enhance the stakeholders’ awareness on nutritional risks [9, 10, 44, 45]. The lack of guidelines may be due to the fact that the health systems in low and middle developing countries was overwhelmed for long time by other priorities such as infectious diseases limiting a focus on food and nutrition [39]. However, developing countries for the past decades have experienced epidemiologic transition and landscape, characterized by change in lifestyle due to economic development, urbanization, unhealthy eating habits, and physical inactivity [40–42]. It is therefore high time now for the health sector to integrate nutritional illness risks into the consumption habit in especially street food policy and guidelines while responding to NCD surge, which is responsible with 33% mortality in Tanzania [40–43, 45, 46].

Concerning food production and preparation environment barriers, the findings indicated fears and concerns of safety of food production and preparation environment as impacting uptake of fruits and vegetables [39, 40]. Safety concerns of contaminated water used for irrigation and areas where vegetables are grown as well as the quality of water used to wash fruits and effectiveness in washing vegetables (preparation) emerged as common concern. It was also observed that vegetables watered with tape water was expensive as compared to those watered with water perceived to be unsafe, such a situation pose a challenge to poor individuals due to affordability issues. The authors understand that the protective role of fruits and vegetable on type2 Diabetes, cardiovascular disease and certain forms of cancer is well known [8, 47]. However, concerns of unsafe environmental for their production and preparation observed in the current study may increase the likelihood of street food consumers to not fully utilize the benefits of fruit and vegetable consumption as a primary prevention measure for NCDs and Diabetes [48, 49]. It was noted that some street food consumers are fearful of consuming vegetables because they think the water used to grow them is contaminated and unsafe. In Tanzania, the high prevalence of Typhoid has been linked with use of water from wells/rivers and frequent eating in the restaurant; such findings may justify fear for fruits and vegetables consumption among street food consumers [50]. It was also noted that some consumers are not ready to consume vegetables and fruits because they perceived that handling, safety of water used to wash fruits, vegetables, and preparation areas are not safe or vegetables are cooked with unhealthy cooking oil. As a result, the findings indicate that most consumers end up eating protein and grain only, even if vegetables or fruits are served on their plates. The fears of contamination of fruits and vegetables by chemicals, parasites, metal or bacteria has been previously documented in studies conducted in Tanzania, Ghana and Ethiopia [50–57]. Minimizing fears of contamination of fruits and vegetables may partly be addressed by enforcing safer production and preparation regulations, having safe production corridors for vegetables and fruits, accreditation of producers and vendors, improving fruits and vegetable vending outlets and interventions focusing on building consumer confidence and addressing misconceptions. The authors further argue that the government need to embrace one-health strategies in responding to NCD challenges especially those related to food production, hygiene, storage and preparation by involving relevant sectors such as agricultural, transport, irrigation, business, bureau of standards etc. [58, 59]. Training of food producers on how to wash and disinfect the vegetables and fruits should be introduced for the good production practices.

Majority of people in Dar-es-Salaam city consume street food daily, they are less likely to consume the WHO recommended amount of fruits and vegetables. Our findings indicate that low consumer income, low consumer knowledge on nutrition, poor eating preference rooted in culture and low vendor capital and limited vendors’ knowledge on nutrition are among the drivers that may impede implementation, uptake and scaling up a healthy plate model among consumers. In this study, it was observed that, with meagre capital, vendors are not able to supply adequate fruits and vegetables and that such endeavour necessitate increasing price for profitability. However, due to low income, most of consumers cannot afford to buy balanced diet even when available and accessible, hence forcing vendors to serve protein and starch rich-plates. Similar barriers to fruits and vegetables consumption have been reported in previous studies. For instance, the social cultural and traditional norms, limited supply of products and materials, individual knowledge, gender, age, income and preference has been widely documented to influence implementation and uptake of healthy food interventions [48–50, 54–56, 60–63]. This imply that implementation and scaling up a healthy plate model necessitate interventions that prioritise both consumers and vendors incentives and consumers health outcomes. The value of interventions that seek to improve consumers’ knowledge of balanced diet and purchasing power of healthy food as well as vendors’ capital and knowledge of preparing healthy food cannot be understated [64–68]. Therefore, measures including economic incentives to vendors and health incentives to consumers, as well as awareness creation are recommended for prevention of NCDs including type2 Diabetes.

Generally, our study present similar results from a review conducted in developing countries to investigate the nutritional contribution of street foods to the diet of people in developing countries. In this study it was concluded that street food contribute significant amount of daily food intake. However, due to high intake of carbohydrate, fats, salt and sugar the street food has a possibility of contributing big role in the development of obesity and non-communicable diseases [38]. Similar findings obtained from our study was also document in another systematic review by Nonato et al., it was documented that street foods have increased risk of causing communicable diseases due to contamination by pathogenic and non-pathogenic microorganisms as well as non-communicable diseases due exposure to due excessive energy intake [45]. Another, global systematic review indicated that poverty, coping strategies to limited access to food and risk factors for the nutritional status of urban poor as main factors which influence healthy eating practices to urban dwellers [69].

### Study limitations

This study met its objective i.e., generating an understanding of the barriers to the introduction of the healthy plate model among street food consumers in three districts of Dar-es-Salaam city. First, study was conducted in only three urban districts of Dar es Salaam City. Although equal geographical representation is not a focus of qualitative inquiries, engagement of participants from districts in the urban City may have limited exploration of the whole range of experiences and challenges that manifest among Consumers and vendors in both urban and rural districts. This may have resulted in underrepresentation of participants’ insights from districts whose challenges may differ from the study sites included. However, inclusion of data from vendors, Consumers from low-income parts of urban districts and food and nutrition administrators aimed to partly address this limitation. Future studies may consider conducting a similar inquiry in rural contexts. Second, the study largely relied on vendors, Consumers and food and nutrition administrators to understand the experiences of healthy plate model. While their accounts provided an insight into their perceptions and experiences of the healthy plate model, insights of fruits and vegetable producers were not examined. Future studies should therefore seek to engage producers of fruits and vegetables to understand the barriers they face in production and packaging of fruits and vegetables in a way that minimise their price in the market but maximising safety and health benefits. Despite the limitations, we strongly argue that the evidence generated in this study will form the basis for designing effective awareness campaign, practical interventions and policy tools with a focus on addressing the issues that street food vendors and consumers face consequently improving uptake and scalability for healthy plate model as a strategy for reducing NCD and Diabetes risks.

## Conclusion

The adoption of the healthy plate model for street food Consumers in Dar es Salaam city continues to be constrained by policy, food production and preparation environment and individual barriers. On the one hand, absence of strategic policy and monitoring practices prioritizing on nutritional illness risks and education guidance for vendors and Consumers and concerns of unsafe environment for production and preparation may affect uptake and scaling up the healthy plate model. On the other hand,consumers’ low knowledge, unhealthy food preference and low income and; limited capital and low knowledge among vendors may also modulate uptake and scaling up of a healthy plate model among consumers. Therefore, promotion of healthy plate model include interventions prioritizing co-construction with all stakeholders considering the barriers each group continue to face. Furthermore, creating positive food and nutrition policy environment, ensuring safety of the food production and preparation, training of stakeholders, and income generation efforts provide useful entry points for successful adoption and scaling up of a healthy plate model. This could consequently contribute towards prevention of diet related NCDs, including Diabetes.

## Supplementary Information

**Additional file 1.**

## Data Availability

The datasets used and/or analysed during the current study available from the corresponding author on reasonable request i.e., after abiding to institution policy.

## Abbreviations

BMI: Body Mass Index
BP: Blood Pressure
FGDs: Focus Group Discussion
KIIs: Key Informants Interview
NCD: Non-communicable Diseases
NIMR: National Institute for Medical Research
WHO: World Health Organization
