# Supplementary material for: Barriers to the implementation, uptake and scaling up of the healthy plate model among regular street food consumers: a qualitative inquiry in Dar-es-Salaam city, Tanzania
Source: BMC Nutr. 2022 Oct 6;8:110. doi: 10.1186/s40795-022-00589-6 (PMC9541087; doi:10.1186/s40795-022-00589-6)
Supplement: Supplementary file 1 — Additional file 1. [file 40795_2022_589_MOESM1_ESM.pdf]

Appendix A

Focus Group Discussion Questions (Street Foods vendors)

1. What do you know about type2 Diabetes (probe on: risk factors, symptoms, prevention strategies, complications)
2. How Diabetes is linked to nutrition values of food consumed by a person (probe on: foods with excess carbohydrate, protein, sugars, salt, fats, less fruits and vegetables)
3. If a person has increased behavioural, metabolic and family history risks for Diabetes, do you think there is a second chance for preventing him/her from disease manifestation? (If Yes/No explain why?)
4. In order to prevent someone from increased metabolic risk factors of Diabetes, what is the best amount of combination of food varieties to be consumed per each plate you sell to your customers (probe on: amount of carbohydrate, protein, sugars, salt, fats, less fruits and vegetables).
5. What are the factors think you will face if the government will instruct the food vendors to serve a plate with the best recommended amount of food varieties i.e. amount of carbohydrate, protein, sugars, salt, fats, less fruits and vegetables (probe on: socio-economic, environmental and cultural factors).

Focus Group Discussion Questions (Street Food Consumers)

1. What do you know about type2 diabetes (probe on: risk factors, symptoms, prevention strategies, complications)
2. How Diabetes is linked to nutrition values of food consumed by a person (probe on: foods with excess carbohydrate, protein, sugars, salt, fats, less fruits and vegetables)
3. If a person has increased behavioural, metabolic and family history risks for diabetes, do you think there is a second chance for preventing him/her from disease manifestation? (If Yes/No explain why?)
4. In order to prevent someone from increased metabolic risk factors of Diabetes, what is the best amount of combination of food varieties to be consumed per each plate you sell to your customers (probe on: amount of carbohydrate, protein, sugars, salt, fats, less fruits and vegetables).
5. What are the factors think you will face if the health service providers will instruct you to consume an healthy food plate i.e. a plate with the best recommended amount of food varieties i.e. amount of carbohydrate, protein, sugars, salt, fats, less fruits and vegetables (probe on: socio-economic, environmental and cultural factors).

Key informant Interview guide (key government stakeholders)

1. Based on your current information what can you comment on trend of behavioural, metabolic and family history risks for Diabetes in this ward/district /county?
2. What is your opinion on the nutritional values of foods served by street food vendors in relation to metabolic risks for Diabetes? (Probe on: amount of carbohydrate, protein, sugars, salt, fats, less fruits and vegetables served).
3. What strategies are in place to ensure that street foods vendors consider the nutritional values of foods they serve to their clients?
4. What are the factors think will affect the strategies for ensuring availability, accessibility and affordability of healthy food plate i.e. a plate with the best recommended amount of food varieties i.e. amount of carbohydrate, protein, sugars, salt, fats, less fruits and vegetables (probe on: policy, socio-economic, environmental and cultural factors)
